# Adherence to Mediterranean Diet and Nutritional Status in Women with Breast Cancer: What Is Their Impact on Disease Progression and Recurrence-Free Patients’ Survival?

**DOI:** 10.3390/curroncol29100589

**Published:** 2022-10-06

**Authors:** Maria Mantzorou, Maria Tolia, Antigoni Poultsidi, Georgios K. Vasios, Dimitrios Papandreou, Stamatios Theocharis, Nikolaos Kavantzas, Andreas Y. Troumbis, Constantinos Giaginis

**Affiliations:** 1 Department of Food Science and Nutrition, School of the Environment, University of the Aegean, GR81400 Myrina, Greece; 2 Department of Radiotherapy, Faculty of Medicine, School of Health Sciences, University of Crete, GR71110 Heraklion, Greece; 3 Department of Surgery, School of Medicine, University of Thessaly, GR41500 Larissa, Greece; 4 Department of Health, College of Natural and Health Sciences, Zayed University, Abu Dhabi P.O. Box 144534, United Arab Emirates; 5 Department of Pathology, School of Medicine, University of Athens, GR11527 Athens, Greece; 6 Department of the Environment, School of the Environment, University of the Aegean, GR81100 Mytilene, Greece

**Keywords:** breast cancer, survival, Mediterranean diet, nutritional status, quality of life, physical activity

## Abstract

**Introduction:** Nutritional status impacts the survival of patients with cancer. There are few studies that investigate the role of nutritional status on breast cancer survival in women with breast cancer, and even fewer regarding the impact of adhering to the Mediterranean diet (MD). The present study aims to assess the nutritional status, MD adherence, physical activity levels and health-related quality of life (HRQOL) in women diagnosed with breast cancer and evaluate these parameters regarding recurrence-free survival. **Methods:** A total of 114 women, aged 35–87 years old, diagnosed with breast cancer in Larissa, Greece, participated in the study. Tumor histopathology was reported, and anthropometric indices were measured by a trained nurse, while questionnaires regarding nutritional status (via mini nutritional assessment), HRQOL via EORTC QLQ-C30, physical activity levels via IPAQ and Mediterranean diet adherence via MedDietScore were administered. The participants were followed-up for a maximum time interval of 42 months or until recurrence occurred. **Results:** A total of 74% of patients were overweight or obese, while 4% of women were undernourished, and 28% were at risk of malnutrition. After 42 months of follow-up, 22 patients (19.3%) had relapsed. The median time to recurrence was 38 months (IQR: 33–40 months) and ranged between 23 to 42 months. Higher levels of MD adherence were significantly associated with lower body mass index (BMI) values, earlier disease stage, smaller tumor size, absence of lymph node metastases and better physical activity levels (*p* < 0.05). Normal nutritional status was significantly associated with higher BMI values and better health-related quality of life (*p* ≤ 0.05). In univariate analysis, patients with higher levels of MD adherence and well-nourished patients had significantly longer recurrence-free survival (*p* < 0.05). In multivariate analysis, MD adherence and nutritional status were independently associated with recurrence-free patients’ survival after adjustment for several confounding factors (*p* < 0.05). **Conclusions:** The impact of MD on time to recurrence is still under investigation, and future interventional studies need to focus on the role of adhering to the MD before and after therapy in survival and breast cancer progression. Furthermore, the present study also highlights the importance of an adequate nutritional status on disease progression, and the need for nutritional assessment, education and intervention in women with breast cancer.

## 1. Introduction

Cancer is one of the most common causes of death worldwide, accounting for one in six deaths, according to the World Health Organization (WHO). Until 2040, the number of new cancer diagnoses is estimated to reach 30.2 million with 16.3 million deaths [1]. In 2022, the American Cancer Society estimated 1.9 million new cancer cases diagnosed and 609,360 cancer deaths for the United States [2]. Breast cancer accounts for one third of diagnoses in the USA for 2022, and is the second leading cause of death due to cancer, with 15% of cancer deaths being due to breast cancer in the USA [2]. Between 2012–2016, the incidence of breast cancer slightly increased by 0.3% per year, due to the diagnosis of local disease [3], as well as higher obesity rates and lower pregnancy rates [4]. It is noteworthy that the incidence of breast cancer is increasing in both pre-menopausal and menopausal women, which intensifies the need for early diagnosis and access to treatment [5].

In terms of patient survival, death rates have decreased by 31% from 1991 to 2018 [6,7] thanks to improved medical care and new healthcare technologies, which facilitate early diagnosis, more effective and personalized treatments and better patient management. In fact, 10-year survival from breast cancer has doubled in the last 40 years in the UK [8]. According to the American Cancer Society, the five-year survival from breast cancer is 90% for all stages [9], while it is 77% for triple-negative cancer of all stages [10], and 41% for the inflammatory breast cancer [10].

The Mediterranean diet (MD) is the most well-studied dietary pattern, and adherence to the MD has been associated with lower risk of chronic diseases [11,12], such as cancer, and breast cancer [13,14,15,16,17]. In fact, the recommendations of the World Cancer Research Fund (WCRF) [18,19] are in line with the MD dietary pattern. Numerous studies have highlighted the protective effect of the MD against breast cancer [20,21,22]. On the other hand, adherence to a Western-type diet has been associated with higher risk of breast cancer [23,24,25].

Regarding the progression of the disease and diet, most studies result in non-significant results [26,27,28,29,30]. In the prospective study by Vrieling et al. [30] with 2522 German post-menopausal women, adherence to a healthy dietary pattern was associated with lower overall mortality (HR 0.74, 95% CI 0.47–1.15, p-trend 0.02) and breast cancer recurrence (HR 0.71, 95% CI 0.48–1.06, p-trend 0.02) in patients at stages I-IIIa [30].

Furthermore, a meta-analysis with 41 cohort studies in breast cancer survivors showed that high intakes of foods that are characteristic of a high quality diet (such as fruits, vegetables, whole grains, nuts, and pulses), and low intakes of red meat (RR 0.74, 95% CI 0.60–0.90, by three studies), as well as adherence to a high quality diet (RR 0.76, 95% CI 0.60–0.95, three studies) are associated with lower mortality [19]. Another meta-analysis with 56 observational studies [31] showed that MD adherence was negatively associated with cancer mortality (RR 0.86, 95% CI 0.81–0.91, 15 studies) and overall mortality (RR 0.92, 95% CI 0.87–0.96, 16 studies).

Malnutrition is a common finding in cancer patients, even at the time of diagnosis. Its incidence varies between 31–87%, depending on the stage, type, treatment and the individual patient [32,33]. Weight loss can occur, as a result of elevated energy needs, low energy intake or impaired absorption of nutrients. In cancer patients, undernutrition may be due to various factors. Inflammation and catabolism because of the tumor can result in muscle wasting and weight loss [34] while tumor gastrointestinal obstruction can impair food intake and absorption, as dysphagia, pain, and vomiting may be present. In addition to this, the side effects of anticancer treatment, such as anorexia, early satiety, nausea, vomiting, and oral and intestinal mucositis with dysphagia, diarrhea, hemorrhoids, anal fissures and modifications in smell and taste affect not only the total energy intake, but also the nutrient absorption, compromising nutritional status. The poor mental health state of cancer patients can affect their energy intake, as well [35,36].

Malnutrition can impact the progress of the disease and the survival of the patients. Studies have shown that weight loss in cancer is associated with poor prognosis, worse quality of life (QoL), lower physical activity level, increased treatment-related adverse effects and reduced tumor response to treatment [33,37]. Weight loss at diagnosis has been associated with shorter failure-free and overall survival (OS), being identified as an independent prognostic factor. However, when patients stop losing weight, they have better OS [38]. Nutritional status is important in breast cancer patients as well, even though few studies have explored its prognostic value [39]. In fact, pre-operative prognostic nutritional index (PNI) in triple negative breast cancer patients is an independent prognostic factor of five-year overall and disease-free survival [40].

Cancer cachexia is usually diagnosed in patients with liver, stomach, colorectal, lung and head and neck cancers [41]. In women with breast cancer, cancer cachexia is rare, but it might be under diagnosed [42]. Studies in patients with bone metastases have highlighted that skeletal muscle mass is affected, revealing a potential association between cancer cachexia and bone metastasis, that is common in advanced cancer [42].

The prognostic role of the nutritional status in different cancer types has been evaluated mainly in retrospective studies, revealing a literature gap for prospective studies, especially in women with breast cancer [39]. Additionally, even though the role of adhering to the Mediterranean diet for cancer prevention has been very well studied [43], few studies have explored the role of this dietary pattern regarding cancer survival [44], and breast cancer survival [27,45].

In view of the above considerations, the aim of the present study is to assess the nutritional status, the adherence to the Mediterranean diet, physical activity and the health-related quality of life in women that were recently diagnosed with breast cancer, and evaluate these parameters regarding recurrence-free patients’ survival. The present study is a three-year prospective study, in women that were recently diagnosed with breast cancer in the University General Hospital of Larissa (Larissa, Greece) and have not yet started chemotherapy or radiotherapy.

## 2. Materials and Methods

### 2.1. Subjects

One hundred and fourteen (114) consecutive women recently diagnosed with breast cancer took part in the present study. Recruitment to the study began at the time the results of the biopsy were given to the patients, between the period November 2017 and September 2018. None of the patients had started treatment. The exclusion criterion for the study was personal history of cancer. The study has been approved by the Bioethics Committee of the University of Thessaly (no 24/12 November 2017) and was in compliance with the declaration of Helsinki of the World Medical Association (52nd WMA General Assembly, Edinburgh, UK). All patient data are confidential, and the study volunteers were informed regarding the aim of the study, as well as the confidentiality of their data, and agreed to voluntarily take part in this study, and signed a consent form. Clarifying instructions were given to the participants about the completion of questionnaires to facilitate accurate answers.

### 2.2. Study Design

Patient information was given by the hospital and the questionnaires were given to the participants during an interview with a trained dietitian and a nurse. Data regarding the histopathologic analysis and TNM stage was collected from the patients’ files in which organ metastases were diagnosed by computed tomography imaging. During the interview, anthropometric indices (weight, height, body mass Index, mid-arm circumference and calf circumference) were measured. Validated questionnaires were used. Mini nutritional assessment (MNA) [46,47,48,49] was used to assess nutritional status. The European Organization for the Research and Treatment of Cancer Quality of Life Questionnaire (EORTC QLQ-C30) [50,51] was used to assess health-related QoL. The Mediterranean diet score (MedDietScore) was used to assess the adherence to the MD during the past five years [52], and the international physical activity questionnaire (IPAQ) was used to assess physical activity levels [53,54].

Anthropometric parameters were measured by a trained dietitian and a trained nurse according to protocol [55,56]. Weight was measured using the same electronic scale, and height was measured using a portable stadiometer. A non-elastic measuring tape was used to measure the mid-arm and calf circumference. The participants were followed-up for a maximum time interval of 42 months or until recurrence was occurred. Recurrence-free survival was defined as the time interval between the date of diagnosis and the date of detection of recurrence or the date of last follow-up without recurrence for breast carcinoma. The patients were followed up every four months by hospital visits and examinations of the patients.

### 2.3. Statistical Analysis

Statistical analysis was performed by Student’s t-test and one-way ANOVA for continuous risk factors found to follow the normal distribution by the use of Kolmogorov–Smirnov test. Chi-square test was used for categorical risk factors. Mann–Whitney non-parametric test was used for non-normally distributed continuous risk factors between two groups, while Kruskal–Wallis non-parametric test was applied for non-normally distributed risk factors between three or more groups. The quantitative non-normally distributed continuous risk factors are presented as median value (interquartile range, IQR), and the qualitative risk factors as absolute or relative frequencies. Long rank test was used to compare the differences between the recurrence curves constructed with the Kaplan–Meier method at univariate level. Cox proportional hazard regression model was developed to evaluate the association between MD adherence and nutritional status and recurrence-free patients’ survival, at multivariate level. In Cox proportional hazard regression models, multiple risk factors (patients’ age and BMI as continuous or categorical risk factors, and histopathological grade of tumor differentiation, tumor size, lymph node metastasis, distant organ metastases, physical activity, and health-related quality of life as categorical risk factors) which have been shown that they can exert confounding effects in recurrence-free patients’ survival were included as potential confounding factors. Differences were considered significant at *p* < 0.05 at a confidence interval (CI) equal to 95%. The statistical analysis of the survey data was performed by SPSS 21.0 program (Statistical Package for Social Sciences, Chicago, IL, USA).

## 3. Results

### 3.1. Evaluation of the Study Population’s Anthropometry, Disease and Lifestyle Characteristics and Recurrence-Free Survival

In total, 114 women, aged 35 to 87 years old (median age 65.5 years, lower-upper quartiles: 57–75 years), took part in the study. The median BMI was 29.7 Kg/m^2^ (lower-upper quartiles: 24.8–32.5 Kg/m^2^), with 74% of women having a BMI classifying them in overweight or obesity.

As far as tumor histopathological type, 80% of patients had ductal breast carcinoma and 20% of women had lobular breast carcinoma. Regarding the grade of tumor differentiation, 28% had high differentiation grade cancer, 65% medium and 7% had low differentiation grade cancer. According to TNM staging, 41% of women had stage I cancer, 40% of women had stage II cancer, 8% of women had stage 3 and the rest 11% of women had stage 4 cancer. According to tumor size (T), 54% of patients were classified as T1, 34% as T2, 5% as T3 and 6% as T4. A total of 50% of women did not have regional lymph node metastasis (N0), 39% of patients were classified as N1, 9% of women were classified as N2 and 2% as N3. As far as distant metastasis is concerned, 89% of women did not have metastatic cancer (M0), while 11% did (M1).

Physical activity levels were directly classified according to IPAQ. In fact, 55.3% had low physical activity levels, 21.9% had moderate physical activity levels and 22.8% had high physical activity levels. The health-related quality of life (EORTC QLQ-C30) median score was 56.2/100 (range 40/100 to 83.0/100). A total of 50.9% of patients had low health-related quality of life (HRQOL, score ˂ 56.2/100) after dichotomization according to the median value.

Regarding MD adherence, according to the MedDietScore, the median score was 26 (Lower-upper quartile: 25–29) and ranged between 17 and 34. The levels of adherence to the MD were categorized into tertiles to low (MedDietScore < 25), moderate (25 ≤ MedDietScore < 29) and high (MedDietScore ≥ 29).

Regarding nutritional status (assessed via MNA), the median score was 25 (Lower-upper quartile: 22.5–26.5) and ranged between 12.5 and 30. Sixty-eight percent (68%) of the patients had a good nutritional status (score > 23), 28% were at risk of malnutrition (17 ≤ score ≤ 23) and 4% of the women were malnourished (score < 17).

After a maximum follow-up of 42 months, 22 patients (19.3%) had relapsed. The median time to recurrence was 38 months (IQR: 33–40 months) and ranged between 23 to 42 months. At 24 months, 5 patients (4.4%) had relapsed and at 36 months, 15 patients (13.2%) had relapsed.

### 3.2. Associations between Examined Characteristics and Mediterranean Diet (MD) Adherence

Higher levels of MD adherence were significantly more frequently observed in patients with lower BMI values (Table 1). Patients with earlier disease stage were significantly more likely to have higher levels of MD adherence (Table 1). Higher MD adherence was significantly more frequently observed in patients with smaller tumor size and absence of lymph node metastasis (Table 1). Patients with higher MD adherence showed significantly better physical activity levels and nutritional status scores and longer recurrence-free survival times (Table 1). MD adherence was not associated with patients’ age, histopathological type and grade of tumor differentiation, presence of organ metastasis and health-related quality of life (via EORTC QLQ-C30) (Table 1).

### 3.3. Associations between Examined Characteristics and Nutritional Status

Better nutritional status was significantly associated with higher BMI values and greater levels of MD adherence (Table 2). Patients with better nutritional status showed a significantly higher prevalence of better health-related quality of life and longer recurrence-free survival times (Table 2). Nutritional status was not associated with patients’ age, histopathological type and grade of tumor differentiation, disease stage, tumor size, lymph node and distant metastases and physical activity levels (Table 2).

### 3.4. Kaplan–Meier Analysis for the Time to Recurrence after Diagnosis

**Figure 1:**
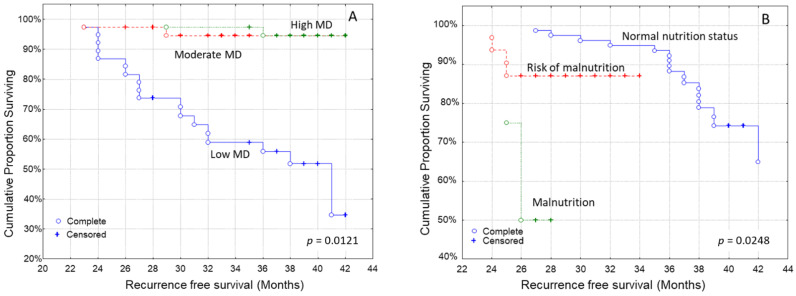
Kaplan–Meier survival analysis stratified according to (**A**) Mediterranean diet (MD) adherence and (**B**) nutritional status in 114 breast carcinoma patients for recurrence-free patients’ survival.

Kaplan–Meier survival curves indicated that patients with higher MD adherence had a longer recurrence-free survival than those who did not follow this dietary pattern to the same extent (Figure 1A, log-rank test, *p* = 0.0121). Additionally, well-nourished patients also had a longer recurrence-free survival time than those at risk of malnutrition or malnourished ones (Figure 1B, log-rank test, *p* = 0.0248).

**Figure 2:**
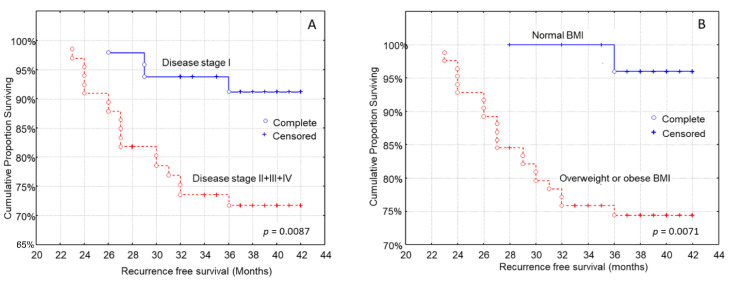
Kaplan–Meier survival analysis stratified according to (**A**) disease stage and (**B**) body mass index (BMI) in 114 breast carcinoma patients for recurrence-free patients’ survival.

Cancer characteristics that impacted recurrence-free survival were tumor size, regional lymph node metastasis, metastatic cancer and disease stage. More to the point, shorter recurrence-free survival was noted in patients with larger tumor size (log-rank test, *p* = 0.0093), patients with regional lymph node metastasis (log-rank test, *p* = 0.0006), metastatic cancer (log-rank test, *p* = 0.0341) and a more advanced stage of disease (Figure 2A, log-rank test, *p* = 0.0087).

Patients’ age (log-rank test, *p* = 0.5849), histopathological type (log-rank test, *p* = 0.7128), and grade of tumor differentiation (log-rank test, *p* = 0.1628) were not associated with the time to recurrence. Patients who were overweight or obese had a shorter time to recurrence than patients with a normal BMI (Figure 2B, log-rank test, *p* = 0.0071).

**Figure 3:**
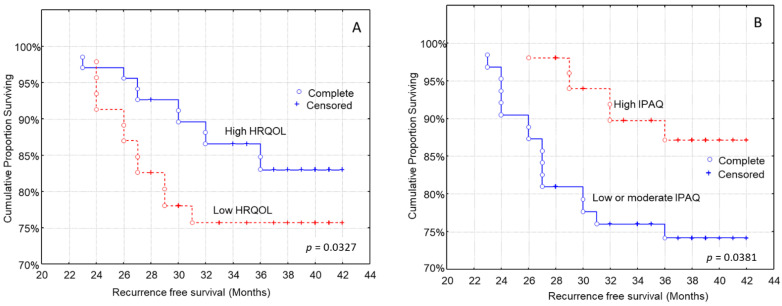
Kaplan–Meier survival analysis stratified according to (**A**) health-related quality of life (HRQOL) and (**B**) international physical activity questionnaire (IPAQ) in 114 breast carcinoma patients for recurrence-free patients’ survival.

The health-related quality of life was also associated with the time to recurrence. Patients with higher EORTC-QLQ-C30 scores had a longer recurrence-free survival (Figure 3A, log-rank test, *p* = 0.0327), than those with lower HRQOL. Moreover, higher physical activity levels were associated with longer recurrence-free survival (Figure 3B, log-rank test, *p* = 0.0381).

### 3.5. Multivariate Analysis for the Time to Recurrence after Diagnosis

In multivariate analysis, high MD adherence was independently associated with longer recurrence-free patients’ survival after adjustment for potential confounding factors (Table 3). Patients’ BMI, histopathological type and nutritional status were also independently associated with recurrence-free patients’ survival after adjustment for confounding factors (Table 3). Patients’ age, histopathological grade, TNM stage, physical activity levels and health-related quality of life did not show any significant impact in multivariate analysis (Table 3).

It should be noted that metastatic disease is such a strong prognostic factor that it may be difficult to completely remove confounding by adjustment, and thus additional multivariate analysis was performed excluding patients with distant metastases and adjusting for the remaining three TNM stages (I, II and III). In the relative Cox regression models developed for MD adherence and nutritional status, almost identical statistics were produced without revealing any difference with the initial models of Table 3 (data not shown). This fact may be ascribed to the low number of M1 cases of the study sample.

## 4. Discussion

In the present prospective study, with a maximum follow-up of 42 months, 19.2% of patients reported recurrence of the disease. In this aspect, 10–41% of breast cancer patients will relapse with metastasis to a different body part, depending on TNM stage and tumor differentiation grade, five years after the end of endocrine therapy [57]. In the meantime, the cumulative risk of recurrence at the 5th and the 20th year after treatment is 6,8% and 21%, respectively [57]. Stage of cancer [58], tumor size and tumor differentiation grade can impact disease prognosis [59]. Even at the early stage of Τ1Ν0, there is a long-term risk of recurrence [60].

Our analysis showed that patients with high MD adherence, and normal nutritional status had a longer recurrence-free survival than those who had lower MD adherence and those at risk of malnutrition or malnourished ones, with the associations between MD adherence, nutritional status and recurrence being independent of confounding factors. Patients who were overweight or obese had a shorter time to recurrence than patients with a normal BMI, according to the Kaplan–Meier analysis.

Shorter recurrence-free survival was also recorded in patients with larger tumor size, patients with regional lymph node metastasis, metastatic cancer and more advanced stage of disease. These findings are in accordance with substantial published evidence supporting the conclusion that an advanced disease stage and its components (higher tumor size, presence of lymph node metastasis and presence of distant organ metastases) are strongly associated with shorter recurrence-free survival in breast cancer [57,58,59,60]. In univariate survival analysis, patients with higher EORTC-QLQ-C30 scores and better physical activity had a longer time to recurrence than those with lower HRQOL and worse physical activity; however, these associations did not remain significant after adjustment for several confounding factors. Patients with high MD adherence showed significantly lower BMI values than those with low MD adherence, as well as better nutritional status.

Higher BMI was also associated with shorter recurrence-free survival, which is in line with the current literature, which shows that higher BMI is associated with worse prognosis [61] in both premenopausal and menopausal women. More specifically, obesity is associated with shorter overall and cancer-specific survival, and being overweight is associated with higher overall survival [62]. Warren et al. [63] found that regional relapse was more common in women who were overweight and obese than those with normal weight. In fact, weight gain after diagnosis is associated with worse prognosis, and patients with obesity have a shorter time to relapse and higher mortality rates [64,65].

Adherence to the Mediterranean diet in Greece has gradually decreased [66,67]. In our study, the median score of MD adherence was 26 with lower-upper quartile: 25–29 which is quite low concerning the international literature, despite the national and international guidelines [66,67]. Adherence to this dietary pattern was associated not only with a longer time to recurrence, but also with better nutritional status. Even though most studies focus on the association between MD and lower risk of cancer, few studies, including the present one, investigate its impact on survival or recurrence.

A recent meta-analysis, which included studies published between 2011–2021 [68] showed that a high-quality diet (via the healthy eating index; HEI-2005, HEI-2015, and the alternative healthy eating Index; AHEI) after diagnosis is associated with 23% lower all-cause mortality, but not cancer-specific mortality. Additionally, an improvement of dietary habits by 10 points, can lower all-cause mortality by 9%. It is important to note that Vrieling et al. [30] in their prospective study found a higher all-cause mortality risk with adherence to an unhealthy diet pattern.

However, Kim et al. [27] did not found a significant association between different questionnaires that assess MD adherence (AHΕΙ, DQIR, RFS, aMED) and breast cancer survival. In that study, diet before and after treatment was not associated with mortality and disease progression. In an earlier study, adherence to a healthy dietary pattern that is similar to the MD, resulted a significantly lower mortality, but no association was found with disease progression (recurrence or breast cancer associated death) in patients with early stages of cancer [69].

Other scores that assess diet quality have failed to find significant associations between diet quality after diagnosis and survival or relapse [29]. However, Weigl et al. [70] note that a healthy balanced dietary pattern is important, nonetheless, for the optimal function of the body. Foods that characterize MD are also sources of bioactive substances with anticancer, anti-inflammatory [71] and antioxidant activity, and their mode of action has been described in pre-clinical studies [72,73].

Nutritional assessment in cancer patients is of utmost importance for the early recognition and treatment of malnutrition [74] yet it is often overlooked [75]. In our study, 28% of patients were at risk of malnutrition and 4% were malnourished. Being malnourished or at risk of malnutrition can shorten recurrence-free survival, according to both our findings, and the literature. Malnutrition in breast cancer patients is an independent factor for shorter survival [39], and more and more studies strengthen the importance of nutritional status in breast cancer prognosis [76,77,78].

Moreover, malnutrition is associated with higher mortality in elderly women with advanced cancer [79], while Bering et al. [80] note that undernutrition in younger patients is rare, but present and can coexist with overweight and obesity, hence nutritional assessment in all patients is important, regardless of body weight. Furthermore, assessment should be repeated frequently, as weight loss before chemotherapy is associated with lower quality of life in breast cancer patients [81]. Personalized nutritional counselling and support is important in order for patients to improve their nutritional status [82,83] and decrease the risk of adverse events and improve prognosis and survival [84].

As far as health-related quality of life is concerned, it was associated with recurrence-free survival, in line with other studies in the literature; however, this association did not remain significant in multivariate analysis. In women over 65 years old with cancer at stages 1–3, quality of life is a prognostic factor for 10-year survival [85] and is negatively affected by recurrence [86]. Patients’ QOL has been improved during the last decades [87]. Even though it worsens at diagnosis, it improves after treatment [88]. Younger patients have lower HRQOL than older women [89] and women at the early stages of cancer have better HRQOL in comparison with those with metastatic cancer [90].

Regarding physical activity levels, one in two women presented low physical activity in our study. Although in this patient group, physical activity is associated with lower mortality [91,92], and better prognosis [93], physical activity levels drop and stay low after treatment [91,94,95,96,97,98]. A recent meta-analysis showed that women with higher physical activity levels have 42% lower all-cause mortality [99]. The mechanism behind the effect of exercise is the improved anticancer activity of the immune system [100]. Exercise can improve myoskeletal disorders after treatment [101] and reduce fatigue levels [102]. Exercise can also improve quality of life [103], self-efficacy and self-confidence [104]. In accordance with the above study, we found that better physical activity was associated with longer recurrence-free patients’ survival; however, this association did not remain significant after adjustment for multiple confounding factors.

The limitations of the present study are the small number of participants, with great heterogeneity regarding characteristics and a relatively short time of follow-up, when taking into account the improved breast cancer survival. In this aspect, future studies focusing on specific patients’ subgroups, e.g., only patients with lobular or ductal breast carcinoma or patients with early disease stage, should be performed. Because the answers of the ΜΝA, EORTC-QLQ-C30 and Mediterranean diet score questionnaires were self-reported, the answers may also have recall bias [105]. The study is also an observational study, hence associations, but not causation can be derived from the data.

In breast cancer, there are many well-known prognostic factors, which, however, were not available in our cohort. Among the most important prognosticators are several tumor receptors such as the estrogen receptor (ER), progesterone receptor and human epidermal growth factor receptor 2 (HER2), as well as the presence of the intraductal component of the tumor, multifocality, and the treatments received by the patients (radiotherapy or chemotherapy). This is another limitation of our study and, therefore, future studies should focus on their potential confounding effects that they may exert on multivariate regression models assessing the impact of MD adherence and nutritional status on recurrence-free patients’ survival.

Moreover, in our study population, a rather high proportion of metastatic breast cancer compared to the existing literature was recorded, which may be ascribed by chance to the small number of patients [57,58,59,60]. By increasing the sample of our study, the proportion of metastatic breast cancer may be reduced, becoming more representative in accordance with the existing literature [57,58,59,60]. Lastly, the short-term recurrence rate of our study population is quite high compared to the existing studies [57,58,59,60]. Accordingly, by increasing the sample of our study, the short-term recurrence rate may be lowered, also becoming more representative and in accordance with the existing literature [57,58,59,60].

The present study is one of the few prospective studies that explores the role of the MD diet on breast cancer prognosis, and we also took into account further parameters such as health-related quality of life and physical activity levels. The role of nutrition, physical activity and quality of life in breast cancer patients is also important for their prognosis. Interventions aimed at facilitating patients in planning and preparing meals can also have a positive effect on nutritional status, through adequate nutrient intake. Breast cancer patients are not considered at high risk for malnutrition, and yet poor nutritional status is present in this group of patients. At the same time, interventions aimed at raising awareness and informing patients about the positive effect of exercise, in an easy-to-understand way, with practical solutions (e.g., experiential seminars) can be planned. Health-related quality of life is another important parameter to be considered by the interdisciplinary team that treats breast cancer patients, and which can be improved with interventions that combine healthy eating and gentle exercise [106,107,108].

## 5. Conclusions

The present study highlights the importance of the nutritional status and the adherence to the MD in the prognosis of young and older patients with breast cancer in different stages of the disease. A high Mediterranean diet adherence and a good nutritional status were significantly associated with a longer recurrence-free survival.

The impact of the MD on disease progression is still under investigation, while future studies are expected to analyze the effect of nutritional interventions in order to increase MD adherence, improving breast cancer survival and prognosis. Our findings regarding the nutritional status in breast cancer progress strengthen the findings of previous studies and highlight the importance and need of nutritional assessment, patient education and intervention for all breast cancer patients.

## Figures and Tables

**Table 1:** Associations between examined characteristics and Mediterranean diet (MD) adherence.

Characteristics, n = 114	Mediterranean Diet (MD) Adherence			
	Low (33.3%)	Medium (33.3%)	High (33.3%)	*p*-Value
Age (years, IQR *)	66.7 (53–72)	64.4 (55–76)	64.2 (57–78)	*p* = 0.119
**BMI (Kg/m^2^, IQR)**	32.5 (27.2–36.9)	28.4 (24.3–32.1)	27.1 (23.5–30.8)	*p* ˂ 0.001
**Histopatological type (n, %)**				*p* = 0.682
Ductal breast carcinoma	30 (78,9)	29 (76.3)	32 (84.2)	
Lobular breast carcinoma	8 (21.1)	9 (23.7)	6 (15.8)	
**Tumor grade of differentiation (n, %)**				*p* = 0.062
High	0 (0.0)	6 (15.8)	2 (5.3)	
Medium	25 (65.8)	25 (65.8)	24 (63.2)	
Low	13 (34.2)	7 (18.4)	12 (31.6)	
**Tumor stage (n, %)**				*p* = 0.008
Stage I	1 (2.6)	19 (50.0)	27 (71.0)	
Stage II	21 (55.3)	14 (36.8)	11 (29.0)	
Stage III + IV	16 (42.1)	5 (13.2)	0 (0.0)	
**Tumor size (n, %)**				*p* = 0.017
T1, ≤2 cm	7 (18.4)	24 (63.2)	31 (81.6)	
T2, >2 cm and ≤5 cm	21 (55.3)	12 (31.6)	6 (15.8)	
T3 + 4, >5 cm	10 (26.3)	2 (5.3)	1 (2.6)	
**Presence of lymph node metastasis (n, %)**				*p* = 0.026
No	12 (31.2)	23 (60.5)	25 (65.8)	
Yes	26 (68.4)	15 (39.5)	13 (34.2)	
**Presence of distant metastasis (n, %)**				*p* = 0.059
No	30 (78.9)	37 (97.4)	35 (92.1)	
Yes	8 (21.1)	1 (2.6)	3 (7.9)	
**Physical activity levels (IPAQ, n, %)**				*p* = 0.015
Low	29 (76.3)	15 (39.5)	19 (50.0)	
Moderate	6 (15.8)	9 (23.7)	10 (26.3)	
High	3 (7.9)	14 (36.8)	9 (23.7)	
**Health-related quality of life (EORTC QLQ-C30, n, %)**				*p* = 0.084
Low	18 (47.4)	25 (65.8)	15 (39.5)	
High	20 (52.6)	13 (34.2)	23 (60.5)	
**Nutritional status score** (median, IQR)	23.5 (20.0–26.0)	24.0 (20.5–28.0)	25.5 (21.5–28.5)	*p* = 0.036
**Recurrence-free survival** (months, IQR)	33.0 (30.0–36.5)	35.5 (33.0–38.5)	38.5 (35.0–41.5)	*p* = 0.001

* Interquartile Range (IQR).

**Table 2:** Associations between examined characteristics and nutritional status according to MNA *.

Characteristics, n = 114	Nutritional Status			
	Well-Nourished Score > 23 (n = 78, 68.4%)	Risk of Malnutrition 17 ≤ Score ≤ 23 (n = 32, 28.1%)	Malnourished Score < 17 (n = 4, 3.5%)	*p*-Value
Age (years, IQR **)	63.5 (57.0–71.5)	64.5 (58.0–71.0)	65.5 (59.5–72.5)	*p* = 0.125
**BMI (Kg/m^2^, IQR)**	32.3 (26.3–33.8)	31.8 (25.2–33,2)	29.7 (24.1–34,4)	*p* = 0.022
**Histopatological type (n, %)**				*p* = 0.195
Ductal breast carcinoma	65 (83.3)	24 (75.0)	2 (50.5)	
Lobular breast carcinoma	13 (16.7)	8 (25.0)	2 (50.5)	
**Tumor grade of differentiation (n, %)**				*p* = 0.408
High	4 (5.1)	3 (9.4)	1 (25.0)	
Medium	52 (66.7)	19 (59.4)	3 (75.0)	
Low	22 (28.2)	10 (31.2)	0 (0.0)	
**Tumor stage (n, %)**				*p* = 0.672
Stage I	35 (44.9)	11 (34.4)	1 (25.0)	
Stage II	28 (35.9)	16 (50.0)	2 (50.0)	
Stage III + IV	15 (19.2)	5 (15.6)	1 (25.0)	
**Tumor size (n, %)**				*p* = 0.450
T1, ≤2 cm	46 (59.0)	15 (46.9)	1 (25.0)	
T2, >2 cm and ≤5 cm	23 (29.5)	14 (43.7)	2 (50.0)	
T3 + 4, >5 cm	9 (11.5)	3 (9.4)	1 (25.0)	
**Presence of lymph node metastasis (n, %)**				*p* = 0.888
No	40 (51.3)	18 (56.2)	2 (50.0)	
Yes	38 (48.7)	14 (43.8)	2 (50.0)	
**Presence of distant metastasis (n, %)**				*p* = 0.737
No	70 (89.7)	28 (87.5)	4 (100.0)	
Yes	8 (10.3)	4 (12.5)	0 (0.0)	
**Physical activity levels (IPAQ, n, %)**				*p* = 0.570
Low	41 (52,6)	19 (59.4)	3 (75.0)	
Moderate	16 (20.5)	8 (25.0)	1 (25.0)	
High	21 (26.9)	5 (15.6)	0 (0.0)	
**Health-related quality of life (EORTC QLQ-C30, n, %)**				*p* = 0.050
Low	44 (56.4)	13 (40.6)	1 (25.0)	
High	34 (43.6)	19 (59.4)	3 (75.0)	
**Mediterranean diet score** (median, IQR)	27.0 (23.0–31.0)	26.0 (22.0–30.0)	26.0 (21.0–29.0)	*p* = 0.046
**Recurrence-free survival** (months, IQR)	36.5 (31.5–42.0)	36.0 (31.0–41.0)	34.5 (29.0–39.5)	*p* = 0.022

* Mini nutritional assessment (MNA). ** Interquartile range (IQR).

**Table 3:** Multivariate analysis assessing the impact of MD adherence and nutritional status to recurrence-free patients’ survival.

Characteristics	Recurrence-Free Survival	
	HR * (95% CI **)	*p*-Value
**Age** (below/over median value)	1.10 (0.38–3.03)	*p* = 0.558
**BMI** (normal weight/overweight and obese)	3.07 (2.12–4.91)	*p* = 0.002
**Histological type** (ductal/lobular)	2.98 (1.48–5.23)	*p* = 0.043
**Histological grade** (high and medium/Low)	1.35 (0.24–2.43)	*p* = 0.312
**Tumor size** (≤2 cm/>2 cm)	0.94 (0.13–5.77)	*p* = 0.748
**Presence of lymph node metastases** (No/Yes)	1.06 (0.20–5.89)	*p* = 0.620
**Presence of distant metastases** (No/Yes)	0.97 (0.21–5.12)	*p* = 0.407
**Physical activity levels** (low/moderate and high)	1.12 (0.32–2.59)	*p* = 0.573
**Health-related quality of life** (low/high)	0.66 (0.21–1.54)	*p* = 0.112
**MD adherence**		*p* = 0.017
Low (reference)	1.00	
Moderate	0.47 (0.18–0.79)	
High	0.39 (0.15–0.72)	
**Nutritional status**		*p* = 0.046
Malnutrition (reference)	1.00	
Risk of malnutrition	0.62 (0.21–1.02)	
Well-nourished	0.57 (0.18–0.97)	

* Hazard ratio: HR. ** CI: Confidence interval.

## Data Availability

The data presented in this study are available on request from the corresponding author.
